# Endoscopic ultrasound-guided vascular intervention for isolated gastric varices using the hydrocoil of an electrically detachable system

**DOI:** 10.1055/a-2541-2131

**Published:** 2025-03-20

**Authors:** Kazunori Nagashima, Yasunori Inaba, Ken Kashima, Yasuhito Kunogi, Fumi Sakuma, Akira Yamamiya, Atsushi Irisawa

**Affiliations:** 1 Department of Gastroenterology, Dokkyo Medical University School of Medicine, Shimotsuga-gun, Japan

In recent years, endoscopic ultrasound (EUS)-guided vascular intervention has been applied to the treatment of isolated gastric varices (iGV) 1 . We perform EUS-guided coil deployment with sclerotherapy, for which ethanolamine oleate is injected as a sclerosant after deploying a 0.035-inch hydrocoil 2 3 . The hydrocoil has an electrically detachable system that allows it to be pulled back during coil deployment. This report describes the first video case demonstrating the usefulness of the coil’s pull-back function during treatment of EUS-guided vascular intervention for iGV.

This video presents a typical case ( Video 1 ). The patient, a 55-year-old man, had alcoholic cirrhosis and giant isolated gastric varices ( Fig. 1 ). Three-dimensional contrast-enhanced computed tomography showed the hemodynamics of the varices, which were fed from the short gastric vein ( Fig. 2 ). After the varices were punctured using a 19-G fine-needle aspiration needle (EZ shot3 plus; Olympus Corp., Tokyo, Japan), a 0.035-inch hydrocoil (Azur; Terumo Corp. Tokyo, Japan) was placed. The delivery sheath and hydrocoil have an electrically detachable system that allows the coil to be pulled back. We checked the blood flow by injecting a contrast medium and using the color Doppler function of EUS. Some additional coils were placed using the pull-back function. A sclerosant (ethanolamine oleate) was injected into the feeder ( Fig. 3 ). The treatment was completed after confirmation that blood flow had ceased ( Fig. 4 ).

Endoscopic ultrasound-guided vascular intervention for isolated gastric varices using the hydrocoil of an electrically detachable system.Video 1

**Fig. 1:**
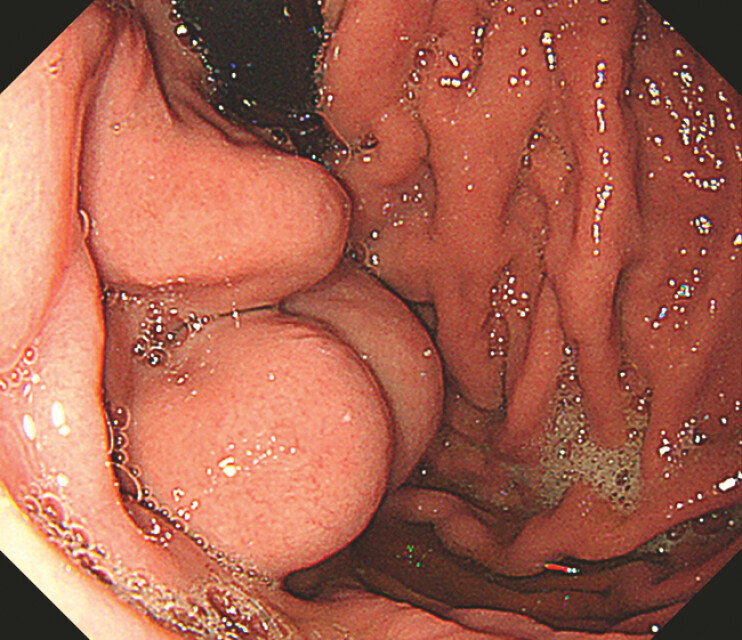
The varices were large and showed strong development.

**Fig. 2:**
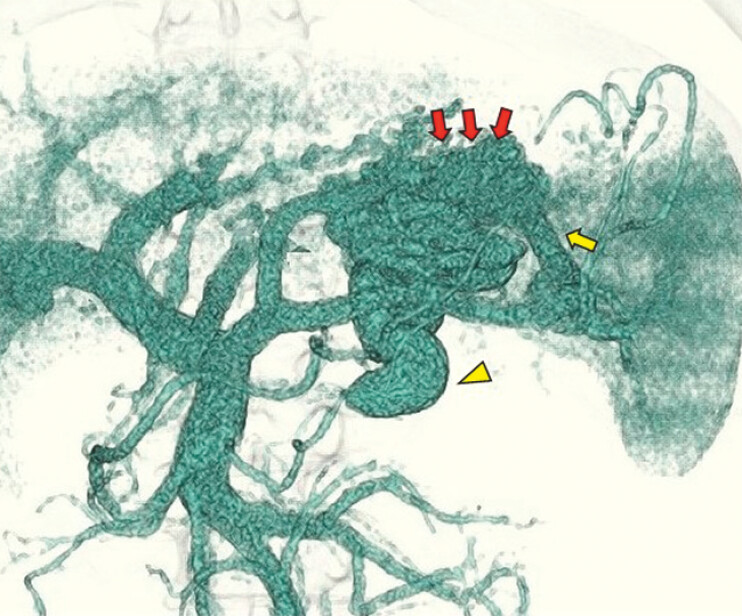
Three-dimensional contrast-enhanced computed tomography revealed the hemodynamics of the varices (red arrows), showing that they were fed from the short gastric vein (yellow arrow) to the renal vein (yellow arrowhead).

**Fig. 3:**
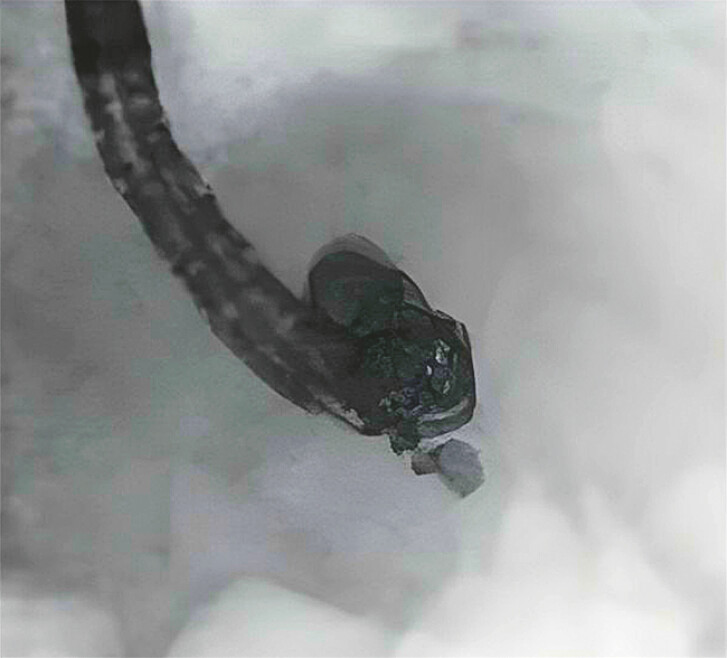
Some additional coils were placed. A sclerosant (ethanolamine oleate) was injected into the feeder.

**Fig. 4:**
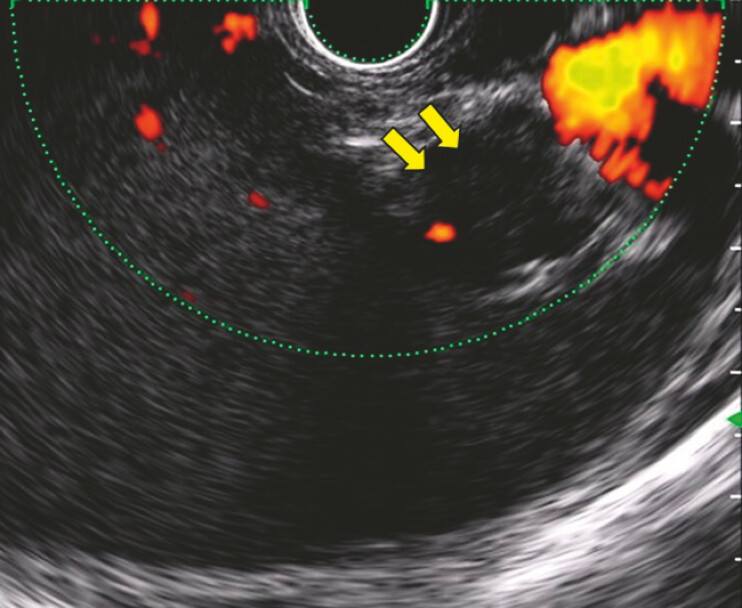
Endoscopic ultrasound revealed that the coils were placed correctly (yellow arrows) and the variceal flow had disappeared.

Wool coils have been used as the standard coil for the EUS-guided vascular intervention 4 . However, for EUS coiling, a 0.035-inch hydrocoil is likely to be considered safer compared with a conventional wool coil, owing to its pull-back function 3 . The special functions of this hydrocoil can be expected to bring high safety and effectiveness not only for treating iGV but also for various EUS-guided vascular interventions.

Endoscopy_UCTN_Code_TTT_1AS_2AB

## Notes

Endoscopy E-Videos https://eref.thieme.de/e-videos
*E-Videos* is an open access online section of the journal *Endoscopy* , reporting on interesting cases and new techniques in gastroenterological endoscopy. All papers include a high-quality video and are published with a Creative Commons CC-BY license. Endoscopy E-Videos qualify for HINARI discounts and waivers and eligibility is automatically checked during the submission process. We grant 100% waivers to articles whose corresponding authors are based in Group A countries and 50% waivers to those who are based in Group B countries as classified by Research4Life (see: https://www.research4life.org/access/eligibility/ ). This section has its own submission website at https://mc.manuscriptcentral.com/e-videos .
